# Estradiol and RSPO3 regulate vertebral trabecular bone mass independent of each other

**DOI:** 10.1152/ajpendo.00383.2021

**Published:** 2022-01-24

**Authors:** Karin H. Nilsson, Jianyao Wu, Karin L. Gustafsson, Maha El Shahawy, Antti Koskela, Juha Tuukkanen, Jan Tuckermann, Petra Henning, Ulf H. Lerner, Claes Ohlsson, Sofia Movérare-Skrtic

**Affiliations:** ^1^Sahlgrenska Osteoporosis Centre, Centre for Bone and Arthritis Research, Institute of Medicine, Sahlgrenska Academy at University of Gothenburg, Gothenburg, Sweden; ^2^Department of Anatomy and Cell Biology, Faculty of Medicine, Institute of Cancer Research and Translational Medicine, University of Oulu, Oulu, Finland; ^3^Institute of Comparative Molecular Endocrinology (CME), University of Ulm, Ulm, Germany; ^4^Region Västra Götaland, Department of Drug Treatment, Sahlgrenska University Hospital, Gothenburg, Sweden

**Keywords:** estrogen, RSPO3, trabecular bone

## Abstract

Osteoporosis is an age-dependent serious skeletal disease that leads to great suffering for the patient and high social costs, especially as the global population reaches higher age. Decreasing estrogen levels after menopause result in a substantial bone loss and increased fracture risk, whereas estrogen treatment improves bone mass in women. RSPO3, a secreted protein that modulates WNT signaling, increases trabecular bone mass and strength in the vertebrae of mice, and is associated with trabecular density and risk of distal forearm fractures in humans. The aim of the present study was to determine if RSPO3 is involved in the bone-sparing effect of estrogens. We first observed that estradiol (E2) treatment increases RSPO3 expression in bone of ovariectomized (OVX) mice, supporting a possible role of RSPO3 in the bone-sparing effect of estrogens. As RSPO3 is mainly expressed by osteoblasts in the bone, we used a mouse model devoid of osteoblast-derived RSPO3 (*Runx2-creRspo3^flox/flox^* mice) to determine if RSPO3 is required for the bone-sparing effect of E2 in OVX mice. We confirmed that osteoblast-specific RSPO3 inactivation results in a substantial reduction in trabecular bone mass and strength in the vertebrae. However, E2 increased vertebral trabecular bone mass and strength similarly in mice devoid of osteoblast-derived RSPO3 and control mice. Unexpectedly, osteoblast-derived RSPO3 was needed for the full estrogenic response on cortical bone thickness. In conclusion, although osteoblast-derived RSPO3 is a crucial regulator of vertebral trabecular bone, it is required for a full estrogenic effect on cortical, but not trabecular, bone in OVX mice. Thus, estradiol and RSPO3 regulate vertebral trabecular bone mass independent of each other.

**NEW & NOTEWORTHY** Osteoblast-derived RSPO3 is known to be a crucial regulator of vertebral trabecular bone. Our new findings show that RSPO3 and estrogen regulate trabecular bone independent of each other, but that RSPO3 is necessary for a complete estrogenic effect on cortical bone.

## Graphical abstract

**Figure:**
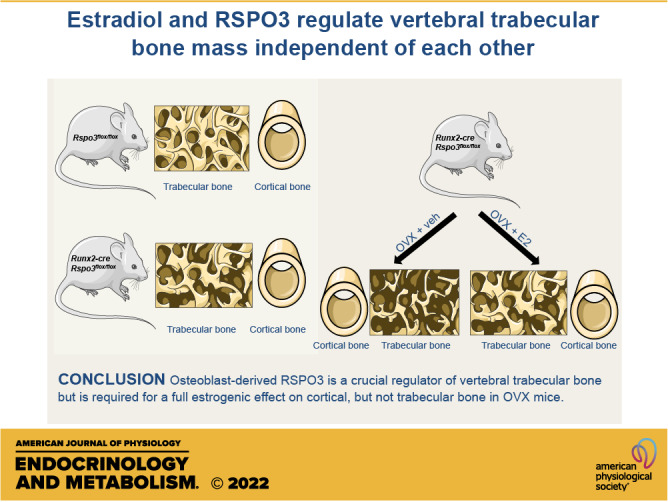

## INTRODUCTION

Osteoporosis is an age-dependent serious skeletal disease that leads to great suffering for the patient and high social costs, especially as the global population reaches higher age (1, 2). Fractures will occur in one in two elderly women and one in four elderly men (3, 4). Decreasing estrogen levels after menopause in women result in a substantial bone loss and increased fracture risk, whereas estrogen treatment improves bone mass and reduces fracture risk in postmenopausal women (5–7). Finding a treatment for osteoporosis caused by estrogen deficiency, without the adverse effects that accompanies treatment with estrogen (6, 8), is of high interest.

WNT proteins is a family of secreted, cysteine-rich proteins, which signals through pathways that are either β-catenin dependent, sometimes called the canonical WNT signaling pathway, or the β-catenin independent pathways, called the noncanonical WNT signaling pathways (9, 10). Modulation of WNT-signaling may influence the cortical bone (11) and/or the trabecular bone (12, 13). Food and Drug Administration has recently approved romosozumab, an antibody against the WNT inhibitor sclerostin, for the treatment of postmenopausal osteoporosis (14, 15). Thus, WNT-signaling is a major regulator of bone mass with clinical relevance for treatment of women with postmenopausal osteoporosis.

R-spondins (RSPOs) is a group of four secreted proteins, RSPO1–4, that binds to the leucin-rich repeat-containing G-protein coupled (LGR) receptors, for example, LGR4–6 (16–18). The binding of RSPOs to the LGR receptor reduces the degradation of Frizzled receptor, leading to an increase in WNT signaling (17, 19–21). Osteoblast-derived RSPO3 increases trabecular bone mass and bone strength in the vertebrae of mice (22). In addition, certain genetic variations in the *RSPO3* locus are associated with trabecular volumetric bone mineral density (vBMD) and risk of distal forearm fractures in humans (22). Previous large-scale human genetic studies reported that the second (after the *WNT16* locus) strongest genetic determinant for fractures at any bone site is located at the *RSPO3* locus (23). These findings indicate that methods targeting osteoblast-derived RSPO3 may be useful for the treatment of osteoporosis.

The aim of the present study was to determine if RSPO3 is involved in the bone-sparing effect of estrogens. In initial studies, we observed that RSPO3 expression in bone is regulated by estrogen and, therefore, we performed a functional study evaluating the role of osteoblast-derived RSPO3 for the stimulatory effects of estrogen on bone mass in ovariectomized (OVX) mice.

## MATERIALS AND METHODS

### Animal Experiments

The animal procedures were approved by the Ethics Committee in Gothenburg, and the animals were cared for according to their guidelines. The animals were housed at a standard animal facility at University of Gothenburg, with a 12-h light/dark period. Food and water were available ad libitum. The mice were euthanized at 17 wk of age using a mix of Ketador/Dexdomitor (Richter Pharma/Orion Pharma), followed by exsanguination and cervical dislocation. Long bones and vertebrae were dissected, fixated in formalin, and stored in ethanol. Soft tissues were collected, weighed, and snap-frozen in liquid nitrogen.

### Generation of *Runx2-creRspo3^flox/flox^* Mice

Mice with cell-specific inactivation of RSPO3 were generated by breeding *Rspo3^flox/flox^* mice (*Rspo3^tm1.1Jcob^*/J, JAX stock 027313, Jackson Laboratories) (24) with the Tg(Runx2-icre)1Jtuc mice, expressing the *cre* construct under the *Runx2* promotor (11, 25), to generate the *Runx2-creRspo3^flox/flox^* mice with a conditional inactivation of RSPO3 in osteoblast-lineage cells, as previously described (22). Tg(Runx2-icre)1Jtuc mice have an unchanged skeletal phenotype compared with wild-type mice (11). As wild-type control, *Rspo3^flox/flox^* littermates were used. The following primers were used to detect the *Rspo3^flox^* allele: forward (5′- TAATGCCCAGGAACTTTTGG-3′) and reverse (5′- GCCTAGAACAGCAACATGGAG-3′). To detect the *cre* construct, a three-primer strategy was used, which previously has been described (11). Briefly, one 5' primer binds to both the transgene and the endogenous gene (5′- CCAGGAAGACTGCAAGAAGG-3′), one 3' primer binds to the *cre* sequence (5′- TGGCTTGCAGGTACAGGAG-3′), and another 3' primer binds to the endogenous *Runx2* (5′- GGAGCTGCCGAGTCAATAAC-3′).

### Estradiol Treatment

**Figure 1.**
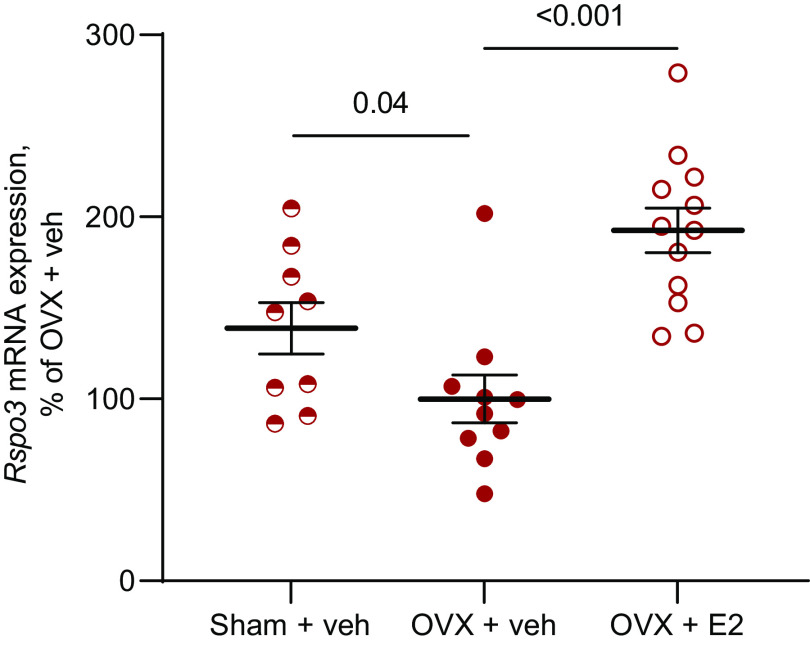
RSPO3 is regulated by estrogen status in wild-type mice. The effect of estrogens on *Rspo3* expression was first evaluated in a descriptive study using wild-type mice. *Rspo3* mRNA expression analyses in cortical diaphyseal bone from 16-wk-old ovariectomized (OVX) mice, treated with either 17β-estradiol (E2, *n* = 12), or vehicle (veh, *n* = 10) for 4 wk, compared with veh-treated, sham-operated mice (*n* = 9). One-way ANOVA followed by Dunnett’s multiple comparison test was used to evaluate the difference in *Rspo3* expression between Sham + veh versus OVX + veh, and OVX + E2 versus OVX + veh mice. A difference was considered significant when *P* < 0.05.

For the mRNA expression in Fig. 1, 12-wk-old wild-type C57BL/6 mice were either ovariectomized (OVX) or sham-operated, treated with a 60-day-slow-release subcutaneous pellet containing either 17β-estradiol (E2, 0.8 µg·kg^−1^·day^−1^, Innovative Research of America) or vehicle (veh). At 16 wk, the mice were euthanized and the long bone dissected, and mRNA extracted. Unrelated data from these mice have previously been published (26).

*Runx2-creRspo3^flox/flox^* and *Rspo3^flox/flox^* mice were OVX at 13 wk of age. Following 1 wk of recovery, the mice were given subcutaneous injections of either 17β-estradiol (E2, 30 µg·kg^−1^·day^−1^, Sigma-Aldrich) or vehicle (veh; Miglyol 812, OmyaPeralta) for 5 days a week for 3 wk. The number of mice in each group was as follows: *Rspo3^flox/flox^*: OVX + veh, 8; OVX + E2, 10; *Runx2-creRspo3^flox/flox^*: OVX + veh, 9; OVX + E2, 9.

### Real-Time Quantitative PCR

Total mRNA was extracted from cortical diaphyseal bone and trabecular vertebral bone using TRIzol (Cat. No. 15596018, Life Technologies) and RNeasy Mini Kit (Cat. No. 74116, Qiagen). The mRNA was reverse transcribed into cDNA (Cat. No. 4374967, Applied Biosystems) and real-time PCR analyses were performed using the StepOnePlus Real-Time PCR System (Thermo Fischer Scientific). The following Assay-on-Demand primer and probe sets were used: *R-spondin 3* (*Rspo3*): Mm01188251_m1; *Dickkopf-1* (*Dkk1*): Mm00438422_m1; *Sclerostin* (*Sost*): Mm00470479_m1; *Transcription factor 7* (*Tcf7*/*Tcf1)*: Mm00493445_m1; and *Axin2*: Mm00443610_m1. The expression of each gene was normalized to the 18S subunit (Cat. No. 4310893E, Thermo Fischer Scientific). The 2^−ΔΔCt^ was used to calculate the relative gene expression.

### Serum Analyses

To assess bone formation markers, serum levels of procollagen type I N-terminal propeptide (P1NP) were measured using a Rat/Mouse EIA kit (Cat. No. AC-33F1, Immunodiagnostic Systems). To measure a marker of bone resorption, serum tartrate-resistant acid phosphatase form 5 b (TRAcP 5 b) was measured using a MouseTRAP ELISA (Cat. No. SB-TR103, Immunodiagnostic Systems).

### Assessment of Bone Parameters

#### Dual-energy X-ray absorptiometry.

Total body bone mineral density (BMD) and lumbar spine BMD were measured using the UltraFocus^DXA^ (Faxitron Bioptics) (27).

#### High-resolution microcomputed tomography.

High-resolution microcomputed tomography (µCT) was used to analyze the proximal femur (SkyScan 1275, Bruker MicroCT) and vertebra L5 (SkyScan 1172, Bruker MicroCT) (11). The cortical region was analyzed in the proximal part of the femur, starting ∼5 mm from the distal growth plate and continuing 200 µm in proximal direction. The trabecular region in the vertebra L5 was analyzed 235 µm from the lower end of the pedicles and continued for ∼229 µm. The data were analyzed using the CTAn software (Bruker MicroCT).

#### Mechanical strength.

Lumbar vertebra L4 was fixated in 4% paraformaldehyde and dehydrated with ethanol. Before testing, ethanol was washed away and the bones were rehydrated in physiological PBS for 24 h. The vertebra was axially loaded with a press head of 2 mm in diameter, with a 1 mm-thick pin through the vertebral foramen to stabilize the sample for testing. The loading speed was 0.155 mm/s. The maximal force was recorded using Instron 3366 testing equipment (Instron) and Bluehill 2 software v2.6 with custom-made Microsoft Excel macros.

### Statistical Analysis

Results are presented as means ± SE. For the statistics in Fig. 1 and Supplemental Table S1 (all Supplemental material is available at https://doi.org/10.6084/m9.figshare.17212013.v1), one-way ANOVA followed by Dunnett’s multiple comparisons tests (GraphPad Prism, version 9.2.0) was used to evaluate the difference in RSPO3 expression between Sham + veh versus OVX + veh, and OVX + E2 versus OVX + veh. For the main experiment evaluating if the effect of E2 treatment differed depending on genotype in OVX mice, two-way ANOVA was used to determine the effect of genotype (*Rspo3^flox/flox^* and *Runx2-creRspo3^flox/flox^*), E2 treatment (veh or E2), and their interaction. A significant interaction term was regarded as a demonstration of affected estrogenic response between *Rspo3^flox/flox^* and *Runx2-creRspo3^flox/flox^* mice. A difference was considered significant when *P* < 0.05.

## RESULTS

### Estrogen Increases *Rspo3* mRNA Expression Levels in Ovariectomized Mice

To determine the possible role of RSPO3 in the bone-sparing effect of estrogens, we first evaluated if RSPO3 expression in bone is dependent on the estrogen status. We observed that *Rspo3* expression in bone was downregulated after ovariectomy (OVX), whereas E2 treatment for 4 wk increased *Rspo3* expression in OVX mice (Fig. 1), indicating that *RSPO3* may be involved in the bone-sparing effect of estrogens. E2 treatment increased expression also of *Tcf7*, but not *Axin2*, in OVX mice (Supplemental Table S1).

**Figure 2.**
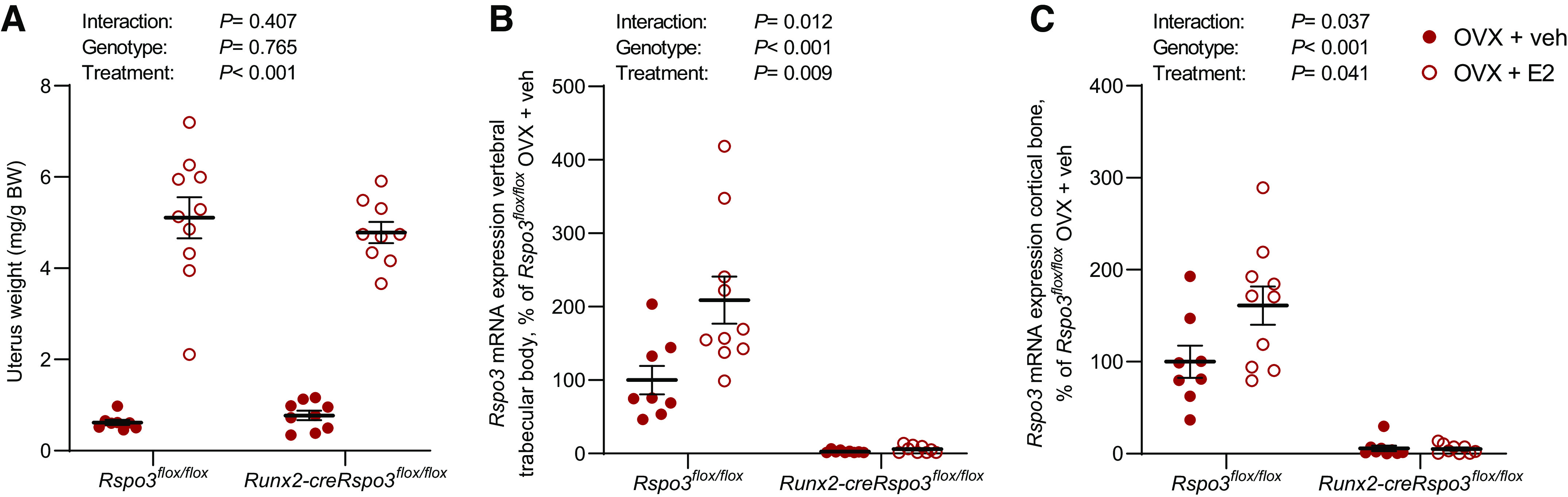
Estrogen increases *Rspo3* mRNA expression levels in ovariectomized mice. The role of osteoblast-derived RSPO3 for the estrogenic response on uterine weight (*A*) and *Rspo3* mRNA expression in vertebral trabecular bone (*B*) and cortical bone (*C*) was evaluated using *Runx2-creRspo3^flox/flox^* mice. *A*: uterus weight per body weight (BW) in *Rspo3^flox/flox^* mice treated with vehicle (veh, *n* = 8) or 17β-estradiol (E2, *n* = 10), and *Runx2-creRspo3^flox/flox^* mice treated with veh (*n* = 9) or E2 (*n* = 9). mRNA expression analyses of *Rspo3* in *Rspo3^flox/flox^* (veh, *n* = 8; E2, *n* = 10) and *Runx2-creRspo3^flox/flox^* mice (veh, *n* = 9; E2, *n* = 9) in trabecular-rich vertebral body (*B*) and cortical bone in the diaphyseal region of femur (*C*). All results refer to 17-wk-old, ovariectomized (OVX) mice. All individual values presented with mean (horizontal line) and SE (vertical line). Two-way ANOVA was used to evaluate the overall interaction effect, genotype effect, and E2 treatment effect. A significant interaction effect demonstrated that the estrogenic response differed between the two genotypes. A difference was considered significant when *P* < 0.05.

We next performed functional studies to determine if RSPO3 is involved in the bone-sparing effect of estrogens. As RSPO3 in the bone tissue is mainly expressed by osteoblasts (22), we used a mouse model with a conditional inactivation of RSPO3 in osteoblast-lineage cells (*Runx2-creRspo3^flox/flox^* mice; see Ref. 22). *Runx2-creRspo3^flox/flox^* mice and littermate control *Rspo3^flox/flox^* mice were ovariectomized and treated with E2 or veh for 3 wk. As expected, the uterus weight was significantly increased following E2 treatment (*P* < 0.001), and there was no difference in the estrogenic response between *Runx2-creRspo3^flox/flox^* mice and *Rspo3^flox/flox^* mice (Fig. 2*A*). We also confirm that the *Rspo3* expression was increased by E2 treatment in both the trabecular-enriched vertebral body (Fig. 2*B*) and in cortical bone in the diaphyseal region of tibia (Fig. 2*C*) of *Rspo3^flox/flox^* mice, whereas marginal *Rspo3* expression was observed in the *Runx2-creRspo3^flox/flox^* mice.

### Osteoblast-Derived RSPO3 Is Dispensable for the Stimulatory Effect of Estrogens on Trabecular Bone

**Figure 3.**
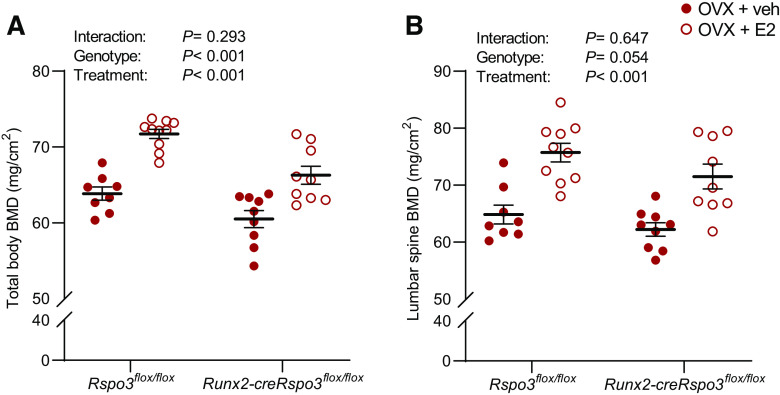
Osteoblast-derived RSPO3 is dispensable for estrogens bone-sparing effect on areal BMD. The role of osteoblast-derived RSPO3 for the estrogenic response on total body areal bone mineral density (BMD; *A*) and lumbar spine areal BMD (*B*) was evaluated using *Runx2-creRspo3^flox/flox^* mice. *A*: total body areal BMD in *Rspo3^flox/flox^* (veh, *n* = 8; E2, *n* = 10) and *Runx2-creRspo3^flox/flox^* (veh, *n* = 9; E2, *n* = 9) mice. *B*: lumbar spine areal BMD in *Rspo3^flox/flox^* (veh, *n* = 8; E2, *n* = 10) and *Runx2-creRspo3^flox/flox^* (veh, *n* = 9; E2, *n* = 9) mice. All results refer to 17-wk-old ovariectomized (OVX) mice. All individual values presented with mean (horizontal line) and SE (vertical line). Two-way ANOVA was used to evaluate the overall interaction effect, genotype effect, and E2 treatment effect. A significant interaction effect demonstrated that the estrogenic response differed between the two genotypes. A difference was considered significant when *P* < 0.05.

**Figure 4.**
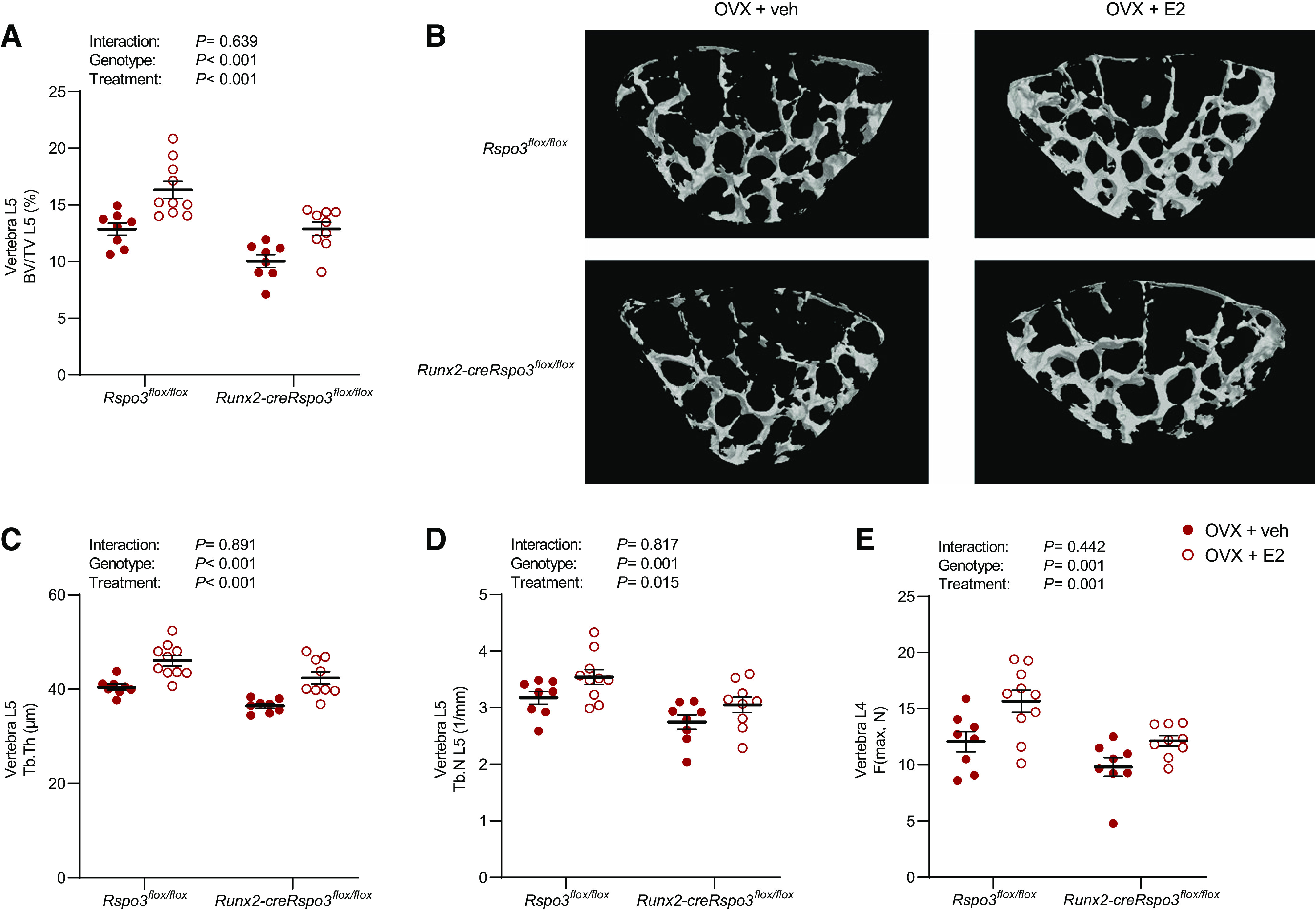
Osteoblast-derived RSPO3 is dispensable for estrogens bone-sparing effect on trabecular bone. The role of osteoblast-derived RSPO3 for the estrogenic response on trabecular bone mass parameters (*A*–*D*) and bone strength in the vertebra (*E*) was evaluated using *Runx2-creRspo3^flox/flox^* mice. *A*: bone volume over tissue volume (BV/TV) in vertebra L5, as measured by µCT, in *Rspo3^flox/flox^* (veh, *n* = 8; E2, *n* = 10) and *Runx2-creRspo3^flox/flox^* (veh, *n* = 9; E2, *n* = 9) mice. *B*: representative 3D µCT images of vertebra L5 in *Rspo3^flox/flox^* mice (*top*) and *Runx2-creRspo3^flox/flox^* mice (*bottom*), treated with either veh (*left*) or E2 (*right*). Trabecular thickness (Tb.Th; *C*) and trabecular number (Tb.N; *D*), in vertebra L5, as measured by µCT, in *Rspo3^flox/flox^* (veh, *n* = 8; E2, *n* = 10) and *Runx2-creRspo3^flox/flox^* (veh, *n* = 9; E2, *n* = 9) mice. *E*: maximal load at failure (N) of vertebra L4 as measured by compression test in *Rspo3^flox/flox^* (veh, *n* = 8; E2, *n* = 10) and *Runx2-creRspo3^flox/flox^* (veh, *n* = 8; E2, *n* = 9) mice. All results refer to 17-wk-old ovariectomized (OVX) mice. All individual values presented with mean (horizontal line) and SE (vertical line). Two-way ANOVA was used to evaluate the overall interaction effect, genotype effect, and E2 treatment effect. A significant interaction effect demonstrated that the estrogenic response differed between the two genotypes. A difference was considered significant when *P* < 0.05.

**Figure 5.**
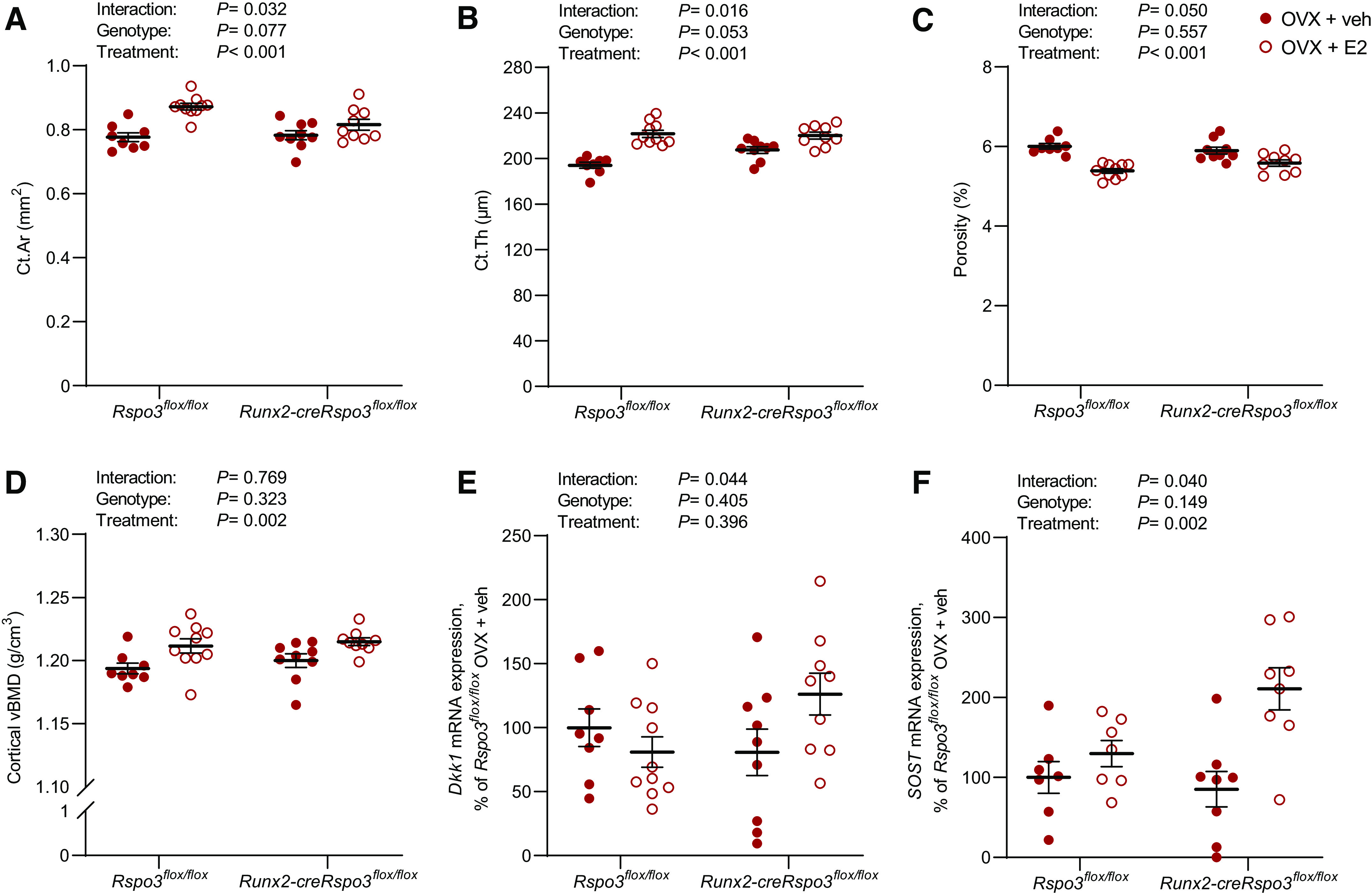
Osteoblast-derived RSPO3 is required for the full stimulatory effect of estrogen on cortical bone thickness. The role of osteoblast-derived RSPO3 for the estrogenic response on cortical bone parameters in the mid-diaphyseal region of femur (*A*–*D*) was evaluated using *Runx2-creRspo3^flox/flox^* mice. Cortical area (Ct.Ar; *A*) and cortical thickness (Ct.Th; *B*) in the mid-diaphyseal region of femur as measured by µCT in *Rspo3^flox/flox^* (veh, *n* = 8; E2, *n* = 10) and *Runx2-creRspo3^flox/flox^* (veh, *n* = 9; E2, *n* = 9) mice. *C*: cortical porosity of femur in the mid-diaphyseal region of femur derived from µCT in *Rspo3^flox/flox^* (veh, *n* = 8; E2, *n* = 10) and *Runx2-creRspo3^flox/flox^* (veh, *n* = 9; E2, *n* = 9) mice. *D*: cortical volumetric BMD (vBMD) of femur derived from µCT in *Rspo3^flox/flox^* (veh, *n* = 8; E2, *n* = 10) and *Runx2-creRspo3^flox/flox^* (veh, *n* = 9; E2, *n* = 9) mice. *E*: mRNA expression analyses of *Dickkopf-1* (*Dkk1*) in diaphyseal cortical bone in *Rspo3^flox/flox^* (veh, *n* = 8; E2, *n* = 10) and *Runx2-creRspo3^flox/flox^* (veh, *n* = 9; E2, *n* = 9) mice. *F*: mRNA expression analyses of *sclerostin* (*Sost*) in diaphyseal cortical bone in *Rspo3^flox/flox^* (veh, *n* = 7; E2, *n* = 7) and *Runx2-creRspo3^flox/flox^* (veh, *n* = 8; E2, *n* = 8) mice. All results refer to 17-wk-old ovariectomized (OVX) mice. All individual values presented with mean (horizontal line) and SE (vertical line). Two-way ANOVA was used to evaluate the overall interaction effect, genotype effect, and E2 treatment effect. A significant interaction effect demonstrated that the estrogenic response differed between the two genotypes. A difference was considered significant when *P* < 0.05.

We first confirmed previous findings that the *Runx2-creRspo3^flox/flox^* mice have substantially reduced trabecular bone mass and bone strength in the vertebra, whereas no effect on cortical bone mass parameters was observed compared with *Rspo3^flox/flox^* mice (Figs. 3*A*, 4, and 5).

BMD was evaluated using DXA, demonstrating that the total body BMD was lower in *Runx2-creRspo3^flox/flox^* mice compared with *Rspo3^flox/flox^* mice (*P* < 0.001). However, E2 treatment increased total areal BMD in both genotypes and no significant interaction effect was observed in the two-way ANOVA, demonstrating similar estrogenic response in the two genotypes (Fig. 3*A*). Also, for the lumbar spine BMD, the RSPO3 inactivation in osteoblast-lineage cells did not affect the bone-sparing effect of E2 treatment (Fig. 3*B*). E2 treatment decreased serum levels of P1NP, and no significant interaction effect was observed using two-way ANOVA (Supplemental Table S2). Serum levels of TRAcP 5 b were not affected by genotype or E2 treatment (Supplemental Table S2).

We next evaluated the estrogenic response specifically in the trabecular bone compartment in the vertebra using µCT. Although the trabecular bone volume over tissue volume (BV/TV) in the vertebra was substantially lower in the *Runx2-creRspo3^flox/flox^* mice compared with the *Rspo3^flox/flox^* mice (Fig. 4, *A* and *B*), the magnitude of the stimulatory effect of E2 treatment did not differ between the two genotypes. We observed similar results with reduced trabecular thickness (Fig. 4*C*) and trabecular number (Fig. 4*D*) in *Runx2-creRspo3^flox/flox^* mice compared with the *Rspo3^flox/flox^* mice and again the magnitude of the estrogenic response was not affected by RSPO3 inactivation in osteoblast-lineage cells. We then evaluated the mechanical strength of vertebra L4 using a standardized compression test of the vertebral body. The maximal load was reduced in the *Runx2-creRspo3^flox/flox^* mice compared with the *Rspo3^flox/flox^* mice, but the stimulatory effect of E2 treatment did not differ between the two genotypes (Fig. 4*E*). Collectively, we observed similar results as a previous report, demonstrating reduced vertebral trabecular bone mass and strength in *Runx2-creRspo3^flox/flox^* mice (22). In addition, the present data demonstrate that the bone-sparing effect of estrogen on trabecular bone mass is independent of osteoblast-derived RSPO3. Thus, estrogen and RSPO3 can regulate trabecular bone mass independent of each other.

### Osteoblast-Derived RSPO3 Is Required for the Full Stimulatory Effect of Estrogen on Cortical Bone Thickness

We next evaluated the role of RSPO3 for the E2 response in cortical bone in OVX mice. The stimulatory effect of E2 on the amount of cortical bone, determined by cortical bone area (Fig 5*A*) and cortical thickness (Fig. 5*B*) in the diaphyseal region of femur, was less pronounced in *Runx2-creRspo3^flox/flox^* mice compared with *Rspo3^flox/flox^* control mice (interaction terms in two-way ANOVA; *P* = 0.032 for cortical area and *P* = 0.016 for cortical thickness). In contrast, the effect of E2 on the two cortical bone property parameters porosity and cortical vBMD did not differ between *Runx2-creRspo3^flox/flox^* mice compared with *Rspo3^flox/flox^* control mice (Fig. 5, *C* and *D*).

In a search for the possible mechanism underlying the role of RSPO3 for the full estrogenic response on the cortical bone thickness, we evaluated the expression of transcripts for WNT signaling modulators. We observed that there was a significant genotype-treatment interaction effect for the expression of transcripts for *Dkk1* and *Sost*, both coding for proteins that are key inhibitory factors of WNT signaling (Fig. 5, *E* and *F*).

## DISCUSSION

Decreasing levels of estrogens result in increased risk of osteoporosis and fractures in postmenopausal women (5, 7, 28). Although RSPO3 is a major regulator of vertebral trabecular bone mass and strength in mice and is associated with trabecular BMD and fracture risk in humans (22), the possible role of RSPO3 for the stimulatory effect of estrogens on bone was unknown. Unexpectedly, we here demonstrate that to achieve a full stimulatory effect of estradiol on cortical, but not trabecular, bone in OVX mice, osteoblast-derived RSPO3 is required.

Activation of WNT signaling in bone increases bone mass and reduces fracture risk supported by numerous experimental animal studies, human genetic association studies, and recently also by human randomized clinical trials demonstrating that an antibody against the WNT signaling inhibitor sclerostin increases bone mass and reduces fracture risk in postmenopausal women (14, 29, 30). However, WNT signaling is also involved in processes underlying carcinogenesis, and it has been proposed that inhibition of sclerostin may result in increased risk of cardiovascular events as indicated by a recent Mendelian randomization study (31). Therefore, it is crucial to delineate bone-specific WNT signaling, avoiding possible off target side effects. Previous research findings show that certain WNT signaling modulating proteins such as WNT16, NOTUM, DKK1, sclerostin, and RSPO3 are highly expressed in bone, and that these may be useful targets for bone-specific modulation of WNT signaling (11, 22, 32–35).

Trabecular and cortical bone may respond differently to treatments, and certain WNT signaling pathways have the capacity to regulate the trabecular and the cortical bone compartments separately (11–13). Previous studies have demonstrated that osteoblast-derived WNT16 and the secreted WNT inhibiting lipase NOTUM are major regulators specifically of cortical bone mass (11, 32, 33, 36). In contrast, WNT10b and the extracellular WNT signaling modulator RSPO3 mainly regulate trabecular bone mass (12, 22). The roles of WNT16 and NOTUM for the stimulatory effects of estrogens on bone mass in OVX mice have previously been characterized (33, 37). In the present study, we determined the possible role of RSPO3 for the beneficial effects of estrogens in the skeleton. We first demonstrated that *Rspo3* expression in bone is downregulated after OVX. These findings indicate that it is possible that RSPO3 is involved in the stimulatory effect of estrogen on bone mass. We next performed functional studies to determine if RSPO3 is involved in the bone-sparing effect of estrogens using E2 treatment of OVX mice, an animal model of estrogen treatment to postmenopausal women (38). As RSPO3 is expressed in osteoblast-lineage cells and not in osteoclasts (22), we used a mouse model with osteoblast-lineage-specific inactivation of RSPO3 and compared the estrogenic response in OVX mice with and without inactivation of RSPO3. In the present study, we first confirmed previous findings that mice with inactivation of RSPO3 in the osteoblast-lineage using *Runx2-creRspo3^flox/flox^* mice have reduced trabecular bone mass and strength in the vertebrae (22). In the previous study, gonadal intact mice were evaluated, whereas in the present study, OVX *Runx2-creRspo3^flox/flox^* mice with and without E2 treatment were evaluated. Collectively, these studies show that the reduced trabecular bone mass and strength in the vertebrae of mice with osteoblast-lineage-specific RSPO3 inactivation is independent of the estrogen status, indicating that the pathways for RSPO3 and E2 treatment to regulate trabecular bone in the vertebrae differ. This notion is supported by our present finding that the estrogenic response on vertebral trabecular bone was not affected by osteoblast-lineage inactivation of RSPO3. Thus, estradiol and RSPO3 regulate vertebral trabecular bone mass independent of each other. In addition, an overall E2 treatment effect significantly decreased P1NP serum levels without interaction effect between the response in *Runx2-creRspo3^flox/flox^* mice and *Rspo3^flox/flox^* control mice, demonstrating that the effect of E2 on circulating levels of this bone turnover marker is not dependent on the presence of RSPO3 in osteoblast-lineage cells.

Unexpectedly, when we evaluated the role of RSPO3 for the estrogenic response in cortical bone in OVX mice, we observed that RSPO3 is required for a full estrogenic response on cortical bone area and thickness. In contrast, the effect of E2 on two cortical bone property parameters, cortical vBMD and porosity, did not differ significantly between *Runx2-creRspo3^flox/flox^* mice compared with *Rspo3^flox/flox^* control mice. Hence, specifically for pathways involved in the regulation of the cortical bone thickness, osteoblast-lineage-specific RSPO3 is required for a full estrogenic response in OVX mice, demonstrating that the role of osteoblast-derived RSPO3 is context dependent. For trabecular bone in the vertebrae, RSPO3 increases bone mass independent of estrogen status while specifically for cortical bone thickness, RSPO3 is required to achieve a full estrogen response in OVX mice.

In a search for the possible mechanism underlying the role of RSPO3 for the full estrogenic response on the cortical bone thickness, we observed that there was a significant genotype-treatment interaction effect for the expression of transcripts for *Sost* and *Dkk1*, suggesting that in the absence of RSPO3 in osteoblast-lineage cells, increased expression of *Sost* and *Dkk1* may partly counteract the stimulatory effect of E2 on cortical bone, whereas in the presence of RSPO3, no such negative feedback loop after E2 treatment may exist. However, the detailed mechanism for the action of RSPO3 to achieve a full estrogenic response on cortical bone thickness in OVX mice remains to be determined.

This study has limitations. First, effects at the *Rspo3* mRNA expression level have not been confirmed at the protein level. Second, RUNX2 is reported to be expressed in chondrocytes, and since RSPO3 also is expressed in a subset of chondrocytes in the growth plate (39), we cannot exclude the possibility that inactivation of RSPO3 expression in chondrocytes also might have contributed to the observed effects. Further mechanistic studies are required to identify differences in the pathways for E2 and RSPO3 to regulate vertebral trabecular bone mass.

In conclusion, although osteoblast-derived RSPO3 is a crucial regulator of vertebral trabecular bone, it is required for a full estrogenic effect on cortical, but not trabecular, bone in OVX mice. Thus, estrogen and RSPO3 regulate vertebral trabecular bone mass independent of each other.

## SUPPLEMENTAL DATA

10.6084/m9.figshare.17212013.v1 Supplemental Tables S1 and S2: https://doi.org/10.6084/m9.figshare.17212013.v1.

## GRANTS

This study was supported by the Swedish Research Council, the Swedish Foundation for Strategic Research, the Swedish state under the agreement between the Swedish Government and the county councils, the ALF-agreement in Gothenburg (Grants 238261, 226481, and 237551), the IngaBritt and Arne Lundberg Foundation, the Royal 80 Year Fund of King Gustav V, the Torsten and Ragnar Söderberg’s Foundation, the Knut and Alice Wallenberg Foundation, the Novo Nordisk Foundation, and the Adlerbertska Research Foundation.

## DISCLOSURES

No conflicts of interest, financial or otherwise, are declared by the authors.

## AUTHOR CONTRIBUTIONS

U.H.L., C.O., and S.M-S. conceived and designed research; K.H.N., J.W., K.L.G., M.E.S., A.K., J. Tuukkanen, J. Tuckermann, P.H., and S.M-S. performed experiments; K.H.N., P.H., U.H.L., C.O., and S.M-S. analyzed data; K.H.N., P.H., U.H.L., C.O., and S.M-S. interpreted results of experiments; K.H.N. prepared figures; K.H.N. drafted manuscript; K.H.N., J.W., K.L.G., M.E.S., A.K., J. Tuukkanen, J. Tuckermann, P.H., U.H.L., C.O., and S.M-S. edited and revised manuscript; K.H.N., C.O. and S.M.-S. approved final version of manuscript.
